# E2 Ubiquitin-Conjugating Enzymes Regulates Dengue Virus-2 Replication in *Aedes albopictus*

**DOI:** 10.3390/microorganisms12122508

**Published:** 2024-12-05

**Authors:** Jiaqi Wang, Xueli Zheng, Xuexue Wang, Daibin Zhong, Guofa Zhou

**Affiliations:** 1 Department of Pathogen Biology, School of Public Health, Southern Medical University, Guangzhou 510515, China; jiaqiwang1121@163.com (J.W.); wxx935325747@163.com (X.W.); 2 Program in Public Health, School of Medicine, University of California, Irvine, CA 92617, USA; dzhong@uci.edu (D.Z.); zhoug@uci.edu (G.Z.)

**Keywords:** E2 ubiquitin-conjugating enzymes, SUMOylation, regulates, dengue virus-2, *Aedes albopictus*

## Abstract

*Aedes albopictus*, a major vector of dengue virus (DENV), has a global distribution. Identifying the key components of the ubiquitin system of *A. albopictus* essential for the replication of viruses could help identify targets for developing broad-spectrum antiviral strategies. This study explores the interaction between E2 ubiquitin-conjugating enzymes (Ubc9) and DENV-2 proteins (NS1, NS5, and E) using cell culture and mosquito models. The replication of DENV-2 and the knockdown efficiency of the Ubc9 gene were assessed through reverse transcription–quantitative polymerase chain reaction. The DENV-2-related protein expression was evaluated via Western blot analysis. The interaction between Ubc9 and DENV E and NS5 proteins was investigated through confocal immunofluorescence and co-immunoprecipitation. RNA interference technology was employed to silence Ubc9 expression in C6/36 cells and in *A. albopictus* mosquitoes. The expression level of Ubc9 in the DENV-2-infected group was 3.5-fold higher than that in the control group. The Ubc9 gene expression in the midgut tissue of the mosquito was significantly upregulated. Transfection of C6/36 and BHK-21 cells with the pAc5.1b-EGFP-Ubc9-HA vector led to the overexpression of Ubc9, which decreased the transcription levels of DENV E and NS1, NS5 proteins. The difference was statistically significant (F = 24.27, *p* < 0.01). The expression levels of DENV NS5 and E proteins significantly decreased after infection with DENV-2, suggesting that the depletion of Ubc9 may limit the replication of DENV-2. Ubc9 regulates DENV-2 replication through SUMOylation in the cells and *A. albopictus*, potentially affecting vector competence and DENV transmission. This is the first study to demonstrate that the Ubc9 of *A. albopictus* plays a significant role in regulating the replication of DENV in both mosquito cells and the mosquito itself. The study results may prove useful in designing appropriate therapeutic approaches for dengue and associated complications.

## 1. Introduction

*Aedes* mosquitoes are the vectors of the dengue virus (DENV), which causes dengue fever and dengue hemorrhagic fever/dengue shock syndrome in humans [1,2]. Approximately 400 million cases of dengue are reported each year globally, putting nearly 3.6 billion people at risk of infection [3]. Although DENV infection presents a serious threat to global public health, no antiviral drugs have yet been developed for its treatment [1,2,3,4]. Therefore, it becomes critical to develop effective antiviral drugs and preventive and control measures against dengue.

DENV is a single-stranded, positive-sense RNA virus of the family Flaviviridae. The DENV genome encodes a single polyprotein precursor, which is processed into three structural proteins (C, prM, and E) and seven nonstructural proteins (NS1, NS2A, NS2B, NS3, NS4A, NS4B, and NS5). The NS5 protein is a viral RNA-dependent RNA polymerase (RdRp) [4,5,6,7]. It subverts the STAT2-mediated antiviral interferon signaling of host innate immunity [8,9,10].

*Aedes albopictus*, distributed worldwide, is one of the principal vectors of DENV [11]. The antiviral defense of mosquitoes is a key determinant of their vector competence, i.e., the ability of mosquitoes to become infected by an arbovirus following an infectious blood meal and subsequently transmit it to a new vertebrate host [12,13,14,15,16]. The innate immune response of mosquitoes is a key determinant of the successful transmission of viruses. Viral infection triggers the activation of innate immunity pathways, including the RNA interference (RNAi) pathway, the Janus kinase–signal transducer and activator of transcription (JAK-STAT), and the Toll and immune deficiency (Imd) pathways, resulting in the transcription of genes responsible for antiviral responses [12,15,16,17,18,19].

Some recent studies have shown that the proteins of both RNA and DNA virus families can be modified by conjugating them with a small ubiquitin-like modifier (SUMO), which facilitates viral replication. SUMOylation is a post-translational modification that regulates protein function by attaching SUMOs to target proteins. It plays a key role in the elimination of viruses by regulating the antiviral immune components of the host [20,21,22]. However, viruses have evolved to hijack the conserved SUMOylation system for their own benefits. They can manipulate the entire process of SUMOylation by interacting with the SUMO pathway. Recent studies have suggested that many viral proteins serve as the targets of the SUMOylation pathway by impacting either their intracellular localization or their biological functions [20,23,24,25]. Moreover, many viral proteins participate in the regulation of the SUMOylation system of the host not only by interacting with the SUMO pathway proteins but also by mimicking the related enzymes [24,25,26]. Notably, various proteins of both DNA and RNA viruses can interact with Ubc9, the hub of protein SUMOylation, leading to the modification of the host protein status or the SUMOylation of viral proteins [24,25]. The target proteins can be SUMOylated mainly by directly recognizing and SUMOylating the Lys residues embedded in a SUMOylation consensus motif by Ubc9 [27,28,29]. Most of the studies to date have focused on flaviviruses for determining the role of SUMOylation during arbovirus infection in mammalian cells [12,30,31,32]. It has been demonstrated that the depletion of Ubc9 could restrict DENV replication [12,30,32]. Zhu et al. reported the SUMOylation of nonstructural protein 5 of the Zika virus (ZIKV) as a host-targeting antiviral strategy. They demonstrated that 2-D08, a SUMOylation inhibitor, decreases the RNA levels of flaviviruses (ZIKV, DENV, yellow fever virus, West Nile virus, and Japanese encephalitis virus) during infection [33]. Su et al. reported that the SUMOylation of the DENV nonstructural 5 (NS5) protein decreases the ubiquitin-mediated degradation of NS5 to promote infection [34]. Stokes et al. reported that the SUMOylation pathway suppresses arbovirus replication in *Aedes aegypti* cells [12]. Weng and Shiao reported a significant increase in the transcription of the Ubc9 of *A. aegypti* in its midgut after a normal blood meal. It has been shown that silencing of the Ubc9 of *A. aegypti* results in a significant inhibition of the production of NS1 (a DENV protein), transcription of the viral genome, and reduction in the viral titer in the mosquito saliva [14]. The transcription of *A. albopictus* Ubc9 was significantly upregulated in the midgut of the mosquito after DENV-2 infection [4]. However, the role (proviral or antiviral) of SUMOylation during arbovirus infection in *A. albopictus* cells and adult mosquitoes is yet to be fully elucidated.

Identifying specific components of the host ubiquitin system essential for the replication of viruses could reveal targets for broad-spectrum antiviral therapies. We previously used isobaric tags for relative and absolute quantification (iTRAQ) to conduct a comparative proteomic analysis between the midgut of DENV-infected *A. albopictus* and a control group and were able to identify 162 different proteins, including the E2 ubiquitin-conjugating enzyme (Ubc9) [4]. This study aims to investigate the interaction between Ubc9 and DENV-2 NS1, NS5, and E proteins in cell lines and within *A. albopictus* in an attempt to obtain a foundation for developing new tools to control the mosquito vectors of DENV.

## 2. Materials and Methods

### 2.1. Cell Culture and Virus Enrichment

*A. albopictus*-derived C6/36 cells were used for the propagation of DENV-2 (GenBank: AF038403.1) [4]. The cells were cultured in the minimal essential medium (RPMI 1640; GIBCO, Invitrogen, Waltham, MA, USA) supplemented with 10% fetal bovine serum and maintained at 28 °C. Cells grown in a 25 cm^3^ culture flask were inoculated with DENV-2 at an MOI of 1. After gentle shaking for 15 min, the culture flask was incubated at 37 °C in 5% CO_2_ for 2 days until clear cytopathic effects were observed. The supernatant was harvested after centrifugation at 800× *g* for 5 min, separated into 1 mL aliquots, and frozen at −80 °C [4]. BHK-21 cells were used for the immunofluorescence and immunoprecipitation assays. The cells were grown at 37 °C in 5% CO_2_ in Dulbecco’s modified Eagle’s medium supplemented with 10% fetal bovine serum and 1% penicillin–streptomycin.

### 2.2. Experimental Infection of Mosquitoes with DENV-2

The Foshan strain of *A. albopictus*, collected from Foshan, Guangdong Province, China, in 1981, was used. The mosquitoes were kept in a microcosm in an insectary at 28 °C, 80% humidity, and a 16–8 h light–dark photoperiod. We used 4–6-day-old female mosquitoes. They were starved for 12 h before beginning the experiments. For the experimental group, the DENV-2 supernatant was collected and the virus supernatant mixed with defibrinated sheep blood (purchasing Guangzhou Angfei Biological Technology Co. Guangzhou City in China; LTD, item number: AFX0001) at a ratio of 2:1. For the control group, the cell culture supernatant and sterile defibrinated sheep blood were mixed in a ratio of 2:1 to prepare a blood meal. The mixtures were stored at 37 °C for 30 min and fed to the starved female mosquitoes for 1 h. The fully blood-fed female mosquitoes were picked out and raised in a new container with 10% glucose. The infectivity experiment was performed three times [4,15]. The experiment was repeated twice.

### 2.3. Plasmid Construction

The E2 conjugase was amplified from the cDNA of C6/36 cells through RT-PCR (Ubc9, GenBank accession number: 109422124). The amplified E2 fragment was cloned into the pAC5.1b-EGFP insect cell expression vector and pcDNA3.1(+)-EGFP mammalian cell expression vector, followed by induction of the target protein expression. The lentiviral vector pLKO.1-CMV-copGFP-PURO-Ubc9 was used to interfere with Ubc9 gene expression. The use of the pLKO.1-CMV vector requires the insertion of an artificially designed oligonucleotide fragment containing a 21 nt interference fragment designed for the mRNA of the Ubc9 gene between the specific restriction sites in the vector. The interfering sequence was CCGG GCGTAAAGATCATCCCTTTGG CTCGAG CCAAAGGGATGATCTTTACGC TTTTTT. Both the forward and reverse oligonucleotide primers were synthesized by Beijing Qingke Biotechnology Co., Ltd., Beijing, China, (Table S1). The forward and reverse oligonucleotides were ligated with the vector after annealing and were then inserted between the restriction enzyme *AgeI* and *EcoRI* sites of the vector to construct a lentiviral interference vector.

### 2.4. Plasmid Transfection and Virus Infection

The constructed pAC5.1b-EGFP-Ubc9-HA and pcDNA3.1(+)-EGFP-Ubc9-HA were transfected into C6/36 and BHK-21 cells, respectively. For plasmid DNA transfection, the cells were treated with the Lipo3000 transfection reagent, according to the Invitrogen™ manufacturer’s instructions. The transfection medium was replaced with a fresh, complete medium 6 h after transfection. The cells were infected with DENV-2 24–48 h after transfection. Proteins and RNA were collected 24 h after viral infection for Western blot and reverse transcription experiments. The experiment was repeated twice.

### 2.5. RNA Extraction and Reverse Transcription

RNA was extracted using TRIzol for both cell and mosquito samples. For extracting the mosquito sample RNA, 3–5 mosquitoes were placed in 1.5 mL enzyme-free EP tubes containing 0.5 mL of TRIzol. The tissue was ground using a homogenizer on an ice box for 5 min and centrifuged at 10,000× *g* for 4 min at 4 °C. After centrifugation, the supernatant was transferred to a new EP tube containing 0.1 mL of chloroform. The EP tube was covered and shaken vigorously up and down for approximately 15 s, incubated at room temperature for about 5 min, and placed in a precooled centrifuge (4 °C) and centrifuged at 12,000× *g* for 15 min. The supernatant was transferred to a new 1.5 mL EP tube. The same volume of isopropanol was added, and the mixture was shaken upside down and incubated at room temperature. The supernatant was carefully poured out, and TRIzol and precooled 75% ethanol (diluted with water containing diethylpyrocarbonate) were added to the EP tube in a 1:1 ratio (volume). The precipitate and the tube wall were washed, and the solution was centrifuged at 8000× *g* at 4 °C for 5 min. The supernatant in the EP tube was removed. The tube mouth was opened to allow the RNA precipitate to dry naturally. After moderate air drying, 30 μL of RNase-free water was added to resuspend the precipitate, and the RNA samples were stored at −80 °C for standby application.

The cells were gently rinsed with PBS twice, followed by the addition of 1 mL of TRIzol. The cells were then incubated for 5 min. Cell RNA was extracted according to the above steps. The RNA samples were reverse-transcribed into cDNA using the GoScript Reverse Transcriptase System Reverse transcription kit. The cDNA samples were stored at −20 °C for standby application [4,11,15].

### 2.6. Quantitative Real-Time PCR

Both the RNA and viral RNA copies of mosquitoes and cell samples were quantified by detecting their cDNA using Hieff^®^ Quantitative polymerase chain reaction (qPCR) SYBR^®^ Green Master Mix (YEASEN, Shanghai, China). The reverse transcription–quantitative polymerase chain reaction (RT-qPCR) reaction mixture (per well) contained 10 μL of the SYBR^®^ green master mix, 1 μL of each primer (10 μM) (Table S1), 2 μL of cDNA or the plasmid standard, and 6 μL of RNase-free water. The reaction was performed in a QuantStudioTM Real-Time PCR System (Applied Biosystems, Foster City, CA, USA) under the following conditions: 95 °C for 10 min, 40 cycles of 95 °C for 10 s, and 60 °C for 60 s. The DENV-2 RNA copies from each sample were quantified by comparing the cycle threshold values with the standard curve [15]. The experiment was repeated three times.

### 2.7. Double-Stranded RNA Preparation

RNAi primers were designed through the E-RNAi Web service to introduce the T7 promoter sequence (5′-TAATACGACTCACTATAGGG-3′) in all forward and reverse primers (Table S1) for subsequent RNA synthesis. The target gene fragments were amplified by using Ex Taq DNA polymerase and then recovered by employing a purification kit. The amplified fragments were inserted into the pGEM^®^-T Easy vector and transfected into DH5α competent cells. Positive colony plasmids were screened and identified. Sequencing was performed to confirm that the cDNA was in the frame. The plasmids were treated with restriction enzymes, and the target fragments were recovered by separation and purification on a 1% agarose gel. The fragments were then amplified by Ex Taq DNA polymerase and purified again. To ensure a high-quality PCR product, the purified PCR product was used as a template for the in vitro synthesis of dsRNA using the T7-Scribe™ Transcription kit. The dsRNA precipitate was dried in the laminar flow hood, dissolved in diethylpyrocarbonate with water, and adjusted to the final concentration of 5 μg/μL in a cryogenic refrigerator (−80 °C) for standby application.

### 2.8. RNAi Interference of Ubc9 Gene Expression in C6/36 Cells

The experimental group was categorized into three groups: a blank control group, a negative control group transfected with the negative control NC-shRNA plasmid, and a third group transfected with shRNA samples. Transfection could be performed when the cell density in the C6/36 six-well plate reached 80%. The C6/36 cells were transfected with 1.5 μg of pLKO.1-CMV-copGFP-PURO-Ubc9 and an empty carrier. The transfection methods have been described in the “Plasmid Transfection and Virus Infection” Section 2.4. The expression of fluorescence was observed using a fluorescence microscope 24 h after transfection.

### 2.9. RNAi Interference of Ubc9 Gene Expression in the Midgut of Mosquitoes

We selected 3–5-day-old female mosquitoes and injected them with dsRNA to silence the Ubc9 gene expression in their midguts. Next, the mosquitoes were injected with 1 μg of dsRNA (5 μg/μL) at the lateral side of their thorax using a syringe. PBS was used as the control group and rps7 was used as the internal reference control group. After the injection, the mosquitoes were transferred to a 15 × 10 × 10 cm cylinder topped with a nylon mesh and provided with a 10% glucose solution. They were continually observed under standard rearing conditions at 28 °C and 80% humidity. Their total RNA was collected 3 days after the injection of dsRNA to verify the silencing efficiency of Ubc9 through RT-qPCR. The experiment was repeated three times.

### 2.10. Western Blot Analysis

#### 2.10.1. Cellular Proteins

Adherent cells cultivated in an ice-cold culture box were extracted. They were then precooled with PBS and lysed using a protein lysis buffer in a ratio of 100:1. Next, 200 μL of the lysate was added to each well of a six-well plate, and 1 mL of the lysate was added to a T25 (Falcon 25 cm^2^ Rectangular Canted Neck Cell Culture Flask with Vented Cap). Lysis was performed at 4 °C for 30 min until full contact was achieved between the lysate and the cells. The cells were then scraped off with a scraper, and the lysate was transferred to a new 1.5 mL EP tube.

#### 2.10.2. Tissue Proteins

The lysates were prepared as described above. Whole-body tissues of 3–5 mosquitoes or the midgut tissues of 30–50 mosquitoes were collected in 1.5 mL autoclaved EP tubes. Next, 100 μL of a protein lysis buffer was added, and the tissues were ground using a tissue grinder. When the tissues were sufficiently ground, 100 μL of the lysate was added to a shaker at 4 °C for 30 min until a full contact was achieved between the lysate and the tissue. The lysed cells were then centrifuged at 4 °C (12,000× *g*) for 15 min and a clear liquid protein was obtained. The 10 μL of the extracted protein sample was separated using sodium dodecyl sulfate polyacrylamide gel electrophoresis (SDS–PAGE) and transferred to a polyvinylidene fluoride membrane (Millipore, Burlington, MA, USA). The membranes were blocked for 2 h using 5% bovine serum albumin (BSA) in a 37 °C incubator. Subsequently, the membranes were incubated overnight at 4 °C in an incubation box containing antibodies. The membranes were thoroughly washed in 1× TBST and then incubated with secondary antibodies (HRP-conjugated anti-mouse IgG or HRP-conjugated anti-rabbit IgG diluted in 5% BSA) for 2 h at 37 °C. Antibodies against DENV NS1/NS5 protein, DENV E protein, HA, and GAPDH were used.

### 2.11. Immunofluorescence Assay

BHK-21 cells were seeded in 12-well plates lined with cell slides and transfected with the endotoxin-free plasmid pcDNA3.1(+)-EGFP-Ubc9-HA prepared above. After 24 h of transfection, the cells were inoculated with DENV. After another 24 h of infection, the BHK-21 cells were fixed with 4% paraformaldehyde and permeabilized with 0.1% Triton X-100, followed by blocking with 1% BSA (PBS). The cells were then incubated overnight with primary antibodies (anti-HA antibody and anti-DENV E antibody) at 4 °C, followed by the addition of diluted fluorescent secondary antibodies (Alexa Fluor^®^ 594, Invitrogen) according to the properties of the primary antibodies. The cells were incubated at 37 °C for 1 h. After removing the fluorescent secondary antibody, the slides were washed three times with 1× PBST, for 5 min each time. The slides were blocked with an anti-fluorescence quench agent containing DAPI. The images were observed under a fluorescence microscope [4]. The experiment was repeated three times.

### 2.12. Co-IP Assay

BHK-21 cells were added to a T25 culture flask, marked as the experimental group, and grown to 80% confluency. Transfection of these BHK-21 cells was performed using Lipofectamine 3000 with 3 μg of pcDNA3.1(+)-EGFP-Ubc9-HA plasmid. DENV infection of the cells was noted after 24 h of transfection. For each 0.5 mL of the lysate supernatant (how this lysate supernatant is obtained is described above in “Tissue Proteins”), 40 μL of protein A agarose resuspension was added and incubated for 30–60 min at 4 °C with a rotary shaker. The supernatant was centrifuged at 500× *g* for 5 min at 4 °C and transferred to a new EP tube. Next, 0.5–2 μg of antibody (anti-HA antibodies and anti-DENV-E antibodies) against the tested protein were added to the prebound supernatant. The mixture was incubated for 2 h at 4 °C. Next, 40 μL of protein A agarose was added to each 0.5 mL of the supernatant containing the antibody. The suspension was resuspended, and the mixture was incubated for 2 h at 4 °C or overnight. The supernatant was removed by centrifugation at 500× *g* for 5 min at 4 °C. The protein A agarose medium was washed by resuspension with 800 μL of PBS. This process was performed three times. The protein A agarose medium was resuspended by adding 20–40 μL of the 1× SDS–PAGE loading buffer. It was boiled in a metal bath at 100 °C for 10 min and centrifuged at 500× *g*. Next, an appropriate amount (0.5–2 μg) of the supernatant was removed for the Western blot analysis of the target protein. The experiment was repeated three times.

### 2.13. Statistical Analysis

Normality of the data was assessed using the Kolmogorov–Smirnov test. Differences in gene expression levels, based on the mean CT values, were analyzed with the Mann–Whitney test. The Student’s *t*-test and one-way analysis of variance (ANOVA) was used to compare the average DENV load and gene expression in cell lines and mosquitoes. A *p*-value of <0.05 was considered statistically significant. The analyses were performed using the SPSS statistical program, version 20.0 (SPSS, Chicago, IL, USA).

### 2.14. Ethical Approval

No specific permits were required for the described cell and mosquito experiment. The use of sheep blood by commercial purchase (Guangzhou Angfei Biological Technology Co., Guangzhou city in China; Ltd. Item number: AFX0001) in mosquito blood-feeding was performed in strict accordance with the recommendations in the Guide for the Care and Use of Laboratory Animals of the National Institutes of Health and the guidelines of Southern Medical University on the experimental use of animals. All of the animals were handled according to approved institutional animal care and use committee (IACUC) protocols (#2017–005) of Southern Medical University.

## 3. Results

### 3.1. Upregulation of Ubc9 Expression in the Midgut of A. albopictus After DENV-2 Infection

**Figure 1:**
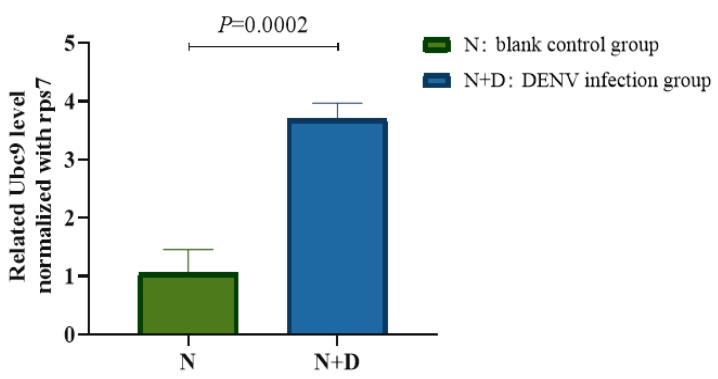
qPCR validation of different expression levels of Ubc9 between the virus-infected group and the control group. A *t*-test was used for statistical analysis, and the results showed that the expression level of Ubc9 in DENV-2-infected *A. albopictus* was significantly upregulated (t = 12.83, *p* < 0.001).

*A. albopictus* was first infected with DENV-2, and then its midgut tissue was dissected on the 14th day after infection. The transcription level of Ubc9 in the midgut tissue was verified through RT-qPCR and was found to be 2.331 ± 0.171 in the infected group and 1.001 ± 0.054 in the control group. Thus, it is clear that the expression level of Ubc9 in the DENV-2-infected group was 3.5-fold higher than that in the control group. Hence, it can be suggested that the expression level of Ubc9 in DENV-2-infected *A. albopictus* was significantly upregulated (t = 12.83, *p* < 0.001) (Figure 1).

### 3.2. High Expression of Ubc9 Protein Inhibits the Expression of DENV-2 NS1 and E

**Figure 2:**
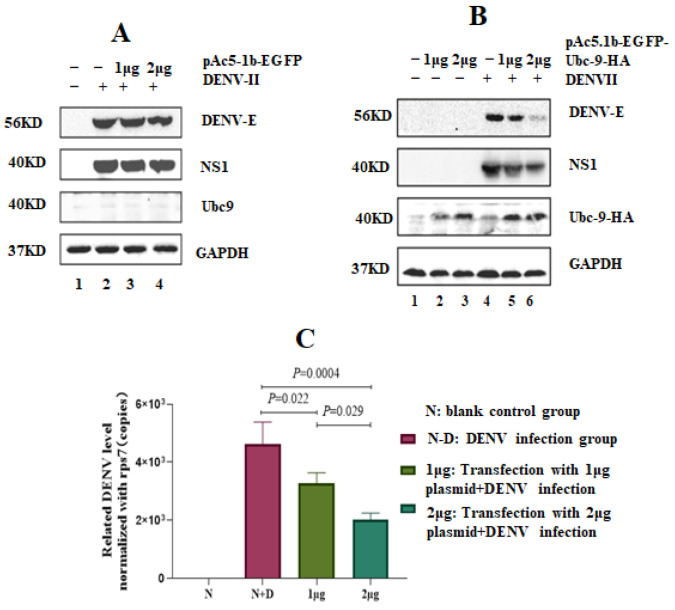
High expression of Ubc9 protein inhibits the expression of DENV NS1 and E in C6/36 cells. (**A**) Expression levels of DENV E and NS1 proteins after the gradient transfection of an empty vector into C6/36 cells. (**B**) Expression levels of DENV E and NS1 proteins after the gradient transfection of Ubc9. When the concentration of pAc5.1b-EGFP-Ubc9-HA in C6/36 cells was increased, the expression of DENV-2 E and NS1 proteins began to decrease. (**C**) The copy number of the DENV transcription level after the gradient transfection of Ubc9 exhibited a downward trend compared with that in control group (*p* < 0.05), which was determined by using ANOVA statistical analysis.

The insect cell expression vector pAc5.1b-EGFP and the constructed recombinant plasmid pac5.1B-EGFP-Ubc9-HA were transfected into C6/36 cells at concentrations of 0, 1, and 2 μg. After 48 h of transfection, the cells were infected with the DENV-2 virus [multiplicity of infection (MOI) = 1)]. Total protein and RNA were extracted after 24 h of infection. A Western blot analysis was conducted, which showed that the pAc5.1b-EGFP vector itself had no impact on the replication of DENV-2 (Figure 2A). However, when the concentration of pAc5.1b-EGFP-Ubc9-HA in C6/36 cells was increased, the expression of DENV-2 E and NS1 proteins began to decrease (Figure 2B). Next, the relative expression level of the DENV virus was calculated using rps7 as the reference gene. A statistical analysis was conducted, which showed that the transcription level of the DENV E protein exhibited a downward trend. The expression level of the virus was statistically significant among all groups (F = 62.26, *p* < 0.001) (Figure 2C).

### 3.3. High Expression of Ubc9 Protein Inhibits the Expression of DENV-2 NS5

**Figure 3:**
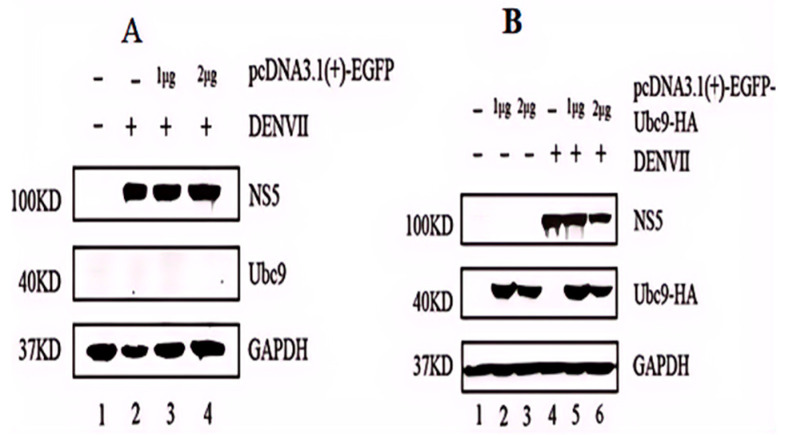
High expression of Ubc9 protein inhibits the expression of DENV NS5 in BHK-21 cells. (**A**) Expression level of DENV NS5 after empty vector transfection into BHK-21 cells. (**B**) Expression levels of DENV NS5 after gradient transfection of Ubc9. ANOVA statistical analysis was conducted, which showed that the transcription level of the DENV E protein exhibited a downward trend compared with that in control group (*p* < 0.05).

BHK-21 cells were transfected with the mammalian cell expression vector pcDNA3.1(+)-EGFP and were marked as the control group. Another group of BHK-21 cells was transfected with the recombinant plasmid pcDNA3.1(+)-EGFP-UBC9-HA and was marked as the experimental group. Both pcDNA3.1(+)-EGFP and pcDNA3.1(+)-EGFP-UBC9-HA were used at a concentration gradient of 0, 1, and 2 μg. After 48 h of transfection, the cells were infected with DENV-2 (MOI = 1). After another 24 h of infection with the virus, total proteins and total RNA of the aforementioned transfected groups were extracted and verified by conducting a Western blot assay. In the blank control group, pcDNA3.1(+)-EGFP had no effect on the replication of DENV-2 (Figure 3A). However, in the experimental group, the protein expression of NS5 decreased with an increase in the transfection DNA concentration of the pcDNA3.1(+)-EGFP-UBC9-HA plasmid (Figure 3B). Hence, it can be suggested that the overexpression of Ubc9 interferes with the expression of DENV NS5, indicating a dose–response relationship.

### 3.4. Co-Immunoprecipitation (CO-IP) Test DENV Env Protein E Interacts with Ubc9 in an Overexpressed System

**Figure 4:**
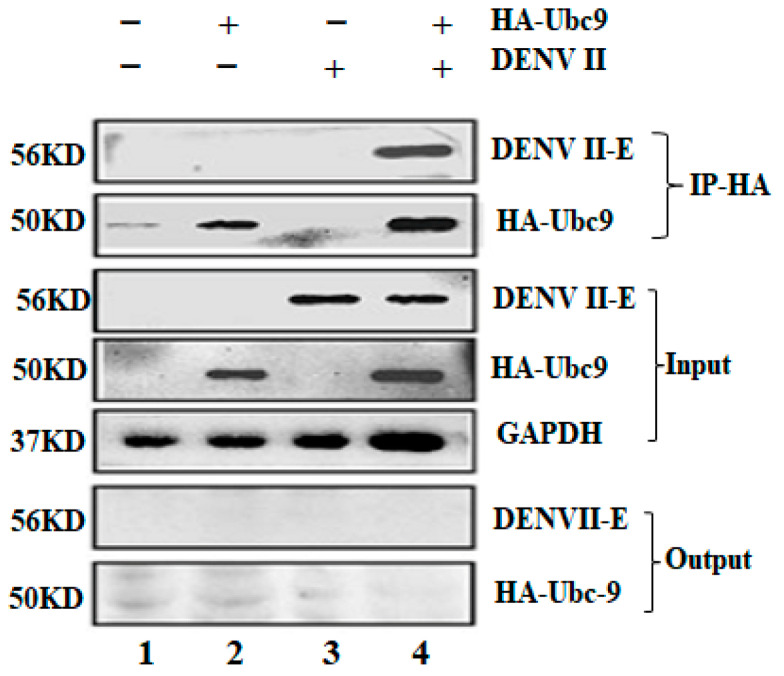
Interaction between Ubc9 and DENV E, as confirmed by CO-IP assay. Lane 1: blank control group; lane 2: 3 μg of pcDNA3.1(+)-EGFP-Ubc9-HA plasmid group; lane 3: DENV infection group; lane 4: 3 μg of pcDNA3.1(+)-EGFP-Ubc9-HA plasmid+DENV infection group. Anti-HA-specific rabbit monoclonal antibody and protein A agarose bead were added in the Co-IP experimental group (IP group). The cell lysate was detected by Western blot with anti-HA antibody. Positive control (Input): Ubc9, the target protein, and the DENV E protein, the protein with which it was predicted to interact, were confirmed to be present in the protein sample. Western blot analysis was performed directly on the cell lysate using anti-HA antibody and anti-DENV E protein-specific antibody to verify the presence of Ubc9 and DENV E proteins in the cell lysate. Output group: Equal amounts of normal rabbit IgG and protein A agar agar beads were added to conduct the Western blot assay of Ubc9 and DENV E proteins, respectively. The experimental results prove that Ubc9 and DENV E interact with each other.

The surface of mature virus particles has DENV env glycoprotein E in the form of homologous trimers. Env protein E comprises three domains: I, II, and III. This protein forms a glycoprotein sheath that connects to receptor molecules on the cell surface and plays a key role in the viral invasion of cells. The interaction between DENV-2 E and Ubc9 was verified in an overexpression system by using the IP group (experimental group), which detected both Ubc9 and DENV E proteins precipitated by Ubc9, while no HA-Ubc9 or DENV E proteins were detected in the output group. The experimental results discussed so far prove that Ubc9 and DENV E interact with each other (Figure 4).

### 3.5. Immunoconfocal Technique Confirmed the Colocalization of Ubc9 and Dengue E Protein

**Figure 5:**
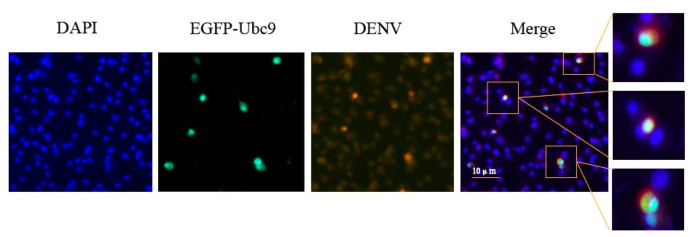
Colocalization of Ubc9 with DENV-2 E protein, as depicted through IF.

The colocalization of Ubc9 and DENV E proteins was verified by using an immunoconfocal technique. The Ubc9 and DENV E proteins were found to be colocalized, indicating an interaction between the two, the experimental results proved that Ubc9 and DENV E interact with each other (Figure 5).

BHK-21 cells were inoculated into a 12-well plate covered with climbing tablets. Each well was transfected with 1.5 μg of pcDNA3.1(+)-EGFP-Ubc9-HA plasmid. After 24 h of transfection, the transfected BHK-21 cells were infected with DENV-2 (MOI = 1). The cells were fixed with 4% paraformaldehyde 24 h after infection. A specific murine monoclonal antibody against the DENV E protein was incubated overnight at 4 °C, followed by incubation with murine fluorescent secondary antibody 594. The Ubc9 and DENV E proteins were found to be colocalized, indicating an interaction between the two. The experimental results prove that Ubc9 and DENV E interact with each other (DAPY showed nuclear staining, EGFP-Ubc9 showed expression of Ubc9, DENV showed expression of DENV E proteins, and the merge showed a merging of Ubc9 with DENV E).

### 3.6. shRNA-Induced Silencing of Ubc9 in C6/36 Cells Decreased the Expression of NS5 and E Proteins in DENV-2

**Figure 6:**
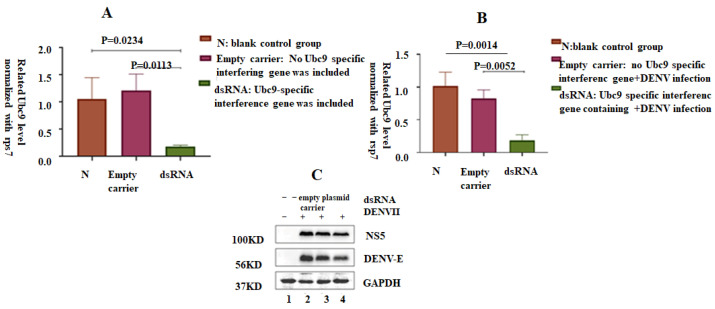
Silencing of Ubc9 inhibited the expression of DENV E and NS5 proteins. (**A**) ANOVA statistical analysis showed significant difference in the expression level of Ubc9 compared with that in the blank control group and the no-load treatment group (*p* < 0.5), in which the C6/36 cells were transfected with an empty lentiviral plasmid (pLKO.1-CMV-copGFP-PURO) and a lentiviral vector comprising a Ubc9-specific shRNA. After transfection for 24–48 h, the C6/36 cells transfected with different plasmids were infected with DENV-2 (MOI = 1). The results show that the expression levels of DENV-2 NS5 and E proteins was lower in experiment group (silencing of Ubc9 group). (**B**) Expression of DENV decreased after silencing the Ubc9 gene. The viral replication level was analyzed by RT-qPCR. Compared with the blank control and no-load treatment groups, the expression level of Ubc9 in the experimental group decreased, and the difference was statistically significant (F = 11.05, *p* < 0.05). (**C**) Expression of DENV E and NS5 proteins decreased after silencing the Ubc9 gene. Western blot results show that after transfecting C6/36 cells with a lentiviral vector containing Ubc9-specific shRNA, the expression levels of DENV NS5 and E protein significantly decreased after infection with DENV-2, suggesting that the depletion of Ubc9 may limit the replication of DENV-2.

Next, the SUMO effect of NS5 and E proteins of DENV-2 was verified. The RNAi technology was used to reduce the expression of Ubc9. An observation of the expression levels of DENV-2 NS5 and E proteins revealed the effect of reducing the level of cell SUMO on viral replication. C6/36 cells transfected with the lentiviral vector containing Ubc9-specific shRNA exhibited decreased expression of Ubc9. ANOVA statistical analysis showed significant difference in the expression level of Ubc9 compared with that in the blank control group and the no-load treatment group (*p* < 0.5), in which the C6/36 cells were transfected with an empty lentiviral plasmid (pLKO.1-CMV-copGFP-PURO) and a lentiviral vector comprising a Ubc9-specific shRNA. After transfection for 24–48 h, the C6/36 cells transfected with different plasmids were infected with DENV-2 (MOI = 1). The total protein and the total RNA of the cells were extracted 24 h after infection. The viral replication level was analyzed by RT-qPCR. Compared with the blank control and no-load treatment groups, the expression level of Ubc9 in the experimental group decreased, and the difference was statistically significant (F = 11.05, *p* < 0.05). Similarly, the lentiviral unloaded plasmid (pLKO.1-CMV-copFGP-PURO) and the lentiviral vector containing Ubc9-specific shRNA were transfected into six-well plate cells and infected with DENV (MOI = 1) 24 h after transfection. Compared with the blank control and unloaded treatment groups, the DENV transcription level in the experimental group decreased, and the difference was statistically significant (F = 24.27, *p* < 0.01). Western blot results showed that after transfecting C6/36 cells with a lentiviral vector containing Ubc9-specific shRNA, the expression levels of DENV NS5 and E protein significantly decreased after infection with DENV-2, suggesting that the depletion of Ubc9 may limit the replication of DENV-2 (Figure 6).

### 3.7. Silencing the Ubc9 Gene in A. albopictus Decreased the Replication Ability of DENV-2

**Figure 7:**
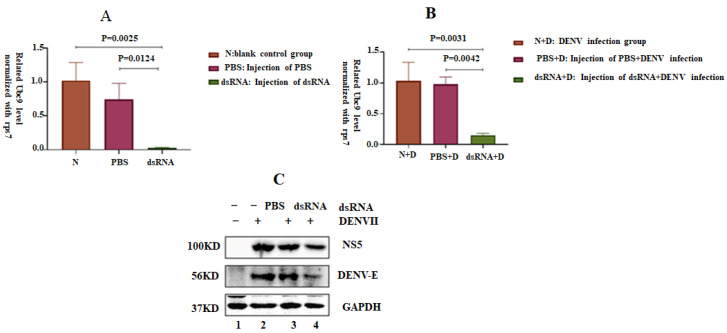
Silencing of the Ubc9 gene in *Aedes albopictus* decreased the replication of DENV proteins. (**A**) RT-qPCR test confirmed the silencing of the Ubc9 gene in *A. albopictus*; a: blank control group, b: PBS injection group, c: dsRNA injection group. Female mosquitoes were injected with approximately 300 nL of dsRNA. The control group was injected with 300 nL of PBS. The total RNA of the mosquitoes was collected after 3 days of injection. The silencing efficiency of Ubc9 was measured by RT-qPCR on day 3. The results show that female *A. albopictus* mosquitoes were injected with dsRNA to silence the Ubc9 gene, the expression level of Ubc9 in the dsRNA experimental group was lower, and the difference was statistically significant (F = 18.78, *p* < 0.05), based on using ANOVA analysis. (**B**) RT-qPCR was used to detect the viral transcription levels of silenced Ubc9 mosquitoes on day 7 of infection with DENV. The DENV transcription level decreased in the experimental group (DENV-infected mosquitoes in the dsRNA-injection). ANOVA statistical analysis showed a significant difference in the copy number level of DENV transcription level compared with that in the blank control group, showing a decrease (F = 21.5, *p* < 0.05). (**C**) Expression levels of DENV NS5 and E proteins in mosquitoes with silenced Ubc9 on the 7th day of DENV infection, as detected by Western blotting.

Female *A. albopictus* mosquitoes were injected with dsRNA to silence the Ubc9 gene. The expression level of Ubc9 in the dsRNA experimental group was lower than that in the control group and the PBS-injection group. The difference was statistically significant (F = 18.78, *p* < 0.05). The mosquitoes were infected with DENV through oral feeding three days after dsRNA injection. The total protein and RNA of mosquitoes were collected on the seventh day of infection. The DENV transcription level decreased, as Western blot analysis showed that the expression levels of DENV E and NS5 proteins decreased after the silencing of the Ubc9 gene (Figure 7C). ANOVA statistical analysis showed a significant difference in the expression level of the DENV transcription level compared with that in the blank control group, showing a decrease (F = 21.5, *p* < 0.05).

## 4. Discussion

Post-translational modification, including phosphorylation, glycosylation, acetylation, ubiquitination, and SUMOylation, is crucial for regulating the function of proteins. SUMOylation involves isopeptide bond formation between the carboxyl group of the modifier and the ε-amino group of a lysine residue in the target protein. Post-translational modification is considered a major regulatory system of protein function that targets many substrates through direct SUMOylation and protein–protein interactions [35,36]. *A. albopictus* was infected with DENV-2. The midgut tissue of the mosquito was dissected on the 14th day of infection. The expression level of Ubc9 in the DENV-2-infected group was 3.5-fold higher than that in the control group, indicating a significant up-regulation in the expression level of Ubc9 in DENV-2-infected *A. albopictus*. A study identified the cellular Ubc9 protein as an interaction partner of the env protein of DENV serotype 2 both in vitro and in vivo [4,32]. Specifically, this interaction depends on residues K51 and K241 of env protein. However, preliminary experiments did not show either association with SUMO-1 or SUMO modification of the env itself. Moreover, some evidence is available to suggest that the overexpression of Ubc9 induces slight changes in the localization of the viral protein during cotransfection [4,32] Viruses have evolved to antagonize the host immune response by interfering with the host ubiquitin-dependent signaling pathways, or by hijacking the cellular ubiquitination machinery, to promote viral replication and pathogenesis [20,37,38,39,40,41,42,43,44,45]. The NS5 protein of flaviviruses is a viral methyltransferase and RNA-dependent RNA polymerase, which is essential for RNA capping, replication of the viral genome, and suppression of the host interferon response [42,46]. Recently, it has been reported that flavivirus-specific SUMO-interacting motif (SIM) in NS5 proteins directs its nuclear localization, and that the SUMOylation of the ZIKA NS5 protein determines its assembly into discrete NBs (nuclear bodies) [5,10,47]. During ZIKV infection, the SUMOylation of NS5 at Lys 252 promotes NS5 NB interactions with STAT2, further disrupting the antiviral promyelocytic leukemia(PML)-STAT2 NBs, promoting PML degradation, and inhibiting the interferon-stimulated gene response, which finally results in the persistent infection of human brain microvascular endothelial cells [10]. SUMO is a reversible post-translational protein modifier. Protein SUMOylation regulates a wide variety of cellular processes and is essential for controlling virus replication. Earlier studies have suggested that the DENV env protein interacts with Ubc9, the sole E2-conjugating enzyme required for protein SUMOylation in mammalian cells [14]. However, little is known about the effect of protein SUMOylation on DENV replication in *A. albopictus*, a major dengue vector.

To determine whether the SUMO modification pathway is involved in DENV replication, we suppressed the SUMOylation system by knocking down Ubc9 expression through RNAi silencing approaches. In both C6/36 and BHK-21 cells transfected with *A. albopictus* Ubc9-specific siRNA, the expression of Ubc9 was substantially suppressed; correspondingly, the replication of the virus also substantially decreased after DENV-2 infection. The *A. albopictus* dsRNA targeting Ubc9 was injected into female mosquitoes through a reverse genetic method, silencing the expression of Ubc9. Hence, it can be suggested that Ubc9-silenced mosquitoes infected with DENV exhibited a significant decrease in the level of virus transcription. Additionally, the production of viral nonstructural protein NS5 and env protein substantially decreased after DENV infection in adult mosquitoes. SUMOylation results in a close connection between the virus and the host.

Thus, a deep understanding of the regulatory mechanisms through which SUMOylation affects viral and antiviral defenses could provide useful insights that enable more effective control of viral infections and the development of SUMOylation-based therapeutics. We constructed the corresponding Ubc9-overexpressed vectors and transfected both C6/36 and BHK-21 cells to up-regulate the Ubc9 gene expression. The protein levels of DENV structural protein E and nonstructural protein NS1 decreased. In the mammalian BHK-21 cells, the nonstructural protein NS5 showed a decreasing trend at the protein level. The overexpression of Ubc9 interfered with the replication of DENV-2, indicating a dose–response relationship. The interaction between Ubc9 and E proteins in BHK-21 cells was confirmed using CO-IP and immunoconfocal techniques. The results suggest that Ubc9 regulates the expression of E and NS5 proteins through a ubiquitination system.

However, silencing the Ubc9 gene in *A. albopictus* decreased the replication ability of DENV-2. There seemed to be a contradiction between the results from the cell level and the mosquito body, which also indicates that the SUMOylation system of regulating dengue virus replication at the cellular and global levels is complex and that they are affected by multiple factors. The exact mechanism is not clear. Wang et al reported the interaction between porcine reproductive and respiratory syndrome virus proteins with the SUMO-conjugating enzyme, which revealed the SUMOylation of nucleocapsid proteins. The overexpression of Ubc9 could inhibit viral genomic replication at an early period of porcine reproductive and respiratory syndrome virus infection [34]. This is consistent with our results at the cellular level. Weng and Shiao reported a significant increase in the transcription of the Ubc9 of A. aegypti in its midgut after a normal blood meal. It has been shown that silencing of the Ubc9 of A. aegypti results in a significant inhibition of the production of NS1 (a DENV protein), transcription of the viral genome and reduction in the viral titer in the mosquito saliva [14]. This is consistent with our experimental results in mosquitoes. Su et al. reported that SUMO modification stabilizes nonstructural protein 5 to support DENV replication. They also found that DENV replication is reduced by silencing the cellular gene Ubc9 [34]. Giraldo et al. reported that the ubiquitination of env protein drives the entry and pathogenesis of the ZIKV. Their data indicate that the ubiquitination of ZIKV E promotes viral replication and that it may be a conserved feature in flaviviruses [48]. Genome-wide short interfering (si) RNA knockdown studies have shown that E3 ubiquitin ligases promote the replication of flaviviruses [37]. Lan et al. reported that the viral subversion of selective autophagy is critical for the biogenesis of viral replication organelles. Infection by many (+)RNA viruses is accompanied by endoplasmic reticulum expansion and membrane remodeling, which result in viral replication organelles, followed by the assembly and secretion of viral progenies. Virus-triggered lipophagy is critical for flaviviral assembly and is driven by ancient ubiquitin protein 1 (Aup1), a lipid droplet-associated protein. Lan et al. also identified Ube2g2, a ubiquitin-conjugating protein that functions as a co-factor for Aup1, as a host-dependency factor [49]. DENV replication is reduced by silencing the cellular gene Ubc9, which encodes the sole E2-conjugating enzyme required for SUMOylation. A comparison of the expression of various NS5 mutants showed that the SUMO acceptor sites are located in the N-terminal domain of NS5. The putative SUMO-interacting motif (SIM) of this domain is crucial for its SUMOylation. The SUMO acceptor sites are located in the N-terminal domain of NS5. A DENV replicon harboring the SUMOylation-defective SIM mutant showed a severe defect in viral RNA replication, supporting the hypothesis that NS5 SUMOylation is necessary for DENV replication. SUMOylation-defective mutants also failed to suppress the induction of STAT2-mediated host antiviral interferon signaling. Furthermore, the SUMOylation of NS5 significantly increased the stability of the NS5 protein [34]. These results are consistent with our findings. Stokes et al. reported that the SUMOylation pathway suppresses arbovirus replication in *A. aegypti* cells. SUMOylation plays an important role, both positively and negatively, in the replication of a wide range of DNA and RNA viruses in mammalian cells, either directly through the SUMOylation of viral proteins or indirectly through the regulation of immune defense [12,20,24,31,43,44,45,50,51].

SUMOylation modulates the antiviral innate immune response of the host. It is involved in regulating the core signal molecules of the innate immune system. It seems that SUMOylation can both enhance and suppress innate antiviral immunity, depending on the substrate specificity and the distinctiveness of the site. Therefore, a thorough understanding of the role of SUMOylation in innate immune response is crucial for preventing and treating virus-associated diseases [2,20,21,22,36,43,44,45,50,51,52,53]. Viral infections are a major health concern worldwide, as they can cause various persistent infections and life-threatening diseases [2,20,21,22,36,43,44,45,50,51,52,53] Targeting SUMOylation is a potential therapeutic approach for treating viral infection-induced diseases. However, a key challenge is the translation of these findings into the development of new antiviral drugs that are suitable for human clinical applications.

Due to the lack of mammalian model organisms and research conditions, this study did not observe how E2 ubiquitin-conjugating enzymes regulate dengue virus-2 replication in mammalian model organisms and failed to provide relevant scientific data for further development of clinical therapeutic dengue drugs. Such research will be carried out if conditions permit.

## 5. Conclusions

This study highlighted the crucial role of Ubc9 in DENV replication, providing a new perspective for understanding the interaction between the DENV and its host, *A. albopictus*. These findings help not only in elucidating the molecular mechanism of the life cycle of DENV but also in providing potential molecular targets for the development of novel antiviral strategies and the use of genetic manipulation of vector mosquitoes to prevent and control mosquito-borne diseases.

## Data Availability

The data sets supporting the results are included within the article and Supplementary Materials. Supplementary Materials can be found in the online version of this article.

## Supplementary Materials

The following supporting information can be downloaded at: https://www.mdpi.com/article/10.3390/microorganisms12122508/s1, Table S1: PCR primers used in this study.
