# Supplementary material for: E2 Ubiquitin-Conjugating Enzymes Regulates Dengue Virus-2 Replication in Aedes albopictus
Source: Microorganisms. 2024 Dec 5;12(12):2508. doi: 10.3390/microorganisms12122508 (PMC11676440; doi:10.3390/microorganisms12122508)
Supplement: Supplementary file 1 [file microorganisms-12-02508-s001.zip › microorganisms-3305952-supplementary.pdf]

Table S1 PCR primers used in this study

Name	Primer (5 '→3')
Name	Primers (3 '→5')
Rps7-F	TCAGTACAAGAAGCTGACCGGA
Rps7-R	TTCCGCGCGCTCACTTATTAGATT
Ubc9-F	ATGTCCGGAATTGCGATCGC
Ubc9-R	TTACTCGGTGGCAGCCATGG
DENV-F	GAAGA CATTGACTGYTGGTGCAA
DENV-R	CGATGTTTCCACGCCCCTTC
